# Development and validation of a gamified sexual and reproductive health education module for adolescent boys in Malaysia

**DOI:** 10.51866/oa.645

**Published:** 2025-01-18

**Authors:** Nazrie Saini, Rosalia Saimon, Razitasham Safii, Jacey Lynn Minoi

**Affiliations:** 1 M.H.Sc (Health Education), Faculty of Medicine & Health Sciences, Universiti Malaysia Sarawak (UNIMAS), Jalan Datuk Mohammad Musa, Kota Samarahan, Sarawak, Malaysia. Email: nazriesaini@gmail.com; 2 PhD, Faculty of Medicine & Health Sciences, Universiti Malaysia Sarawak (UNIMAS), Jalan Datuk Mohammad Musa, Kota Samarahan, Sarawak, Malaysia.; 3 MD, MBBS, MMed (Community Health), Faculty of Medicine & Health Sciences, Universiti Malaysia Sarawak (UNIMAS), Jalan Datuk Mohammad Musa, Kota Samarahan, Sarawak, Malaysia.; 4 PhD, Faculty of Computer Science and Information Technology, Universiti Malaysia Sarawak (UNIMAS) Jalan Datuk Mohammad Musa, Kota Samarahan, Sarawak, Malaysia.

**Keywords:** Adolescent boys, Reproductive health, Health education

## Abstract

**Introduction:**

About one-third of Malaysian adolescents engage in sexual activities before the age of 14 years, putting them at risk for unplanned pregnancies and sexually transmitted diseases. However, implementing sex education in Malaysia remains controversial and challenging. Therefore, this study aimed to develop and validate a newly gamified sexual and reproductive health (SRH) education module *(ReReki)* tailored for Malaysian adolescent boys.

**Methods:**

The *ReReki* module was developed using the analysis, design, development, implementation and evaluation model, based on the theory of planned behaviour. Content validation involved six adolescent health experts, using Russell’s model validity approach and a content validity questionnaire. Games were then designed to complement the Sexual and Reproductive Health (SRH) materials. The gamified *ReReki* module was pre-tested with 20 adolescent boys from a selected school, and the language was refined by a linguistic expert.

**Results:**

The module included five main topics, 29 subtopics and six games. The overall content validity score based on the survey method was 81.8%, while that based on Russell’s model was 79.3%, indicating a high level of validity for the *ReReki* module. One new topic and two subtopics were added, while two subtopics scoring below 70% were flagged for further review. The adolescent boys expressed their acceptance of the gamified SRH content.

**Conclusion:**

All five main topics, along with the 29 subtopics in the *ReReki* module, are suitable and ready for use by adolescent boys in the Malaysian context.

## Introduction

In Malaysia, the youth population becomes a demographic asset when they outnumber the non-productive population in terms of age.^1^ According to the World Health Organization, adolescence is defined as the period from 10 to 19 years of age.^2^ However, trends in sexual and reproductive behaviours among this age group tends to be riskier. In Malaysia, about one-third of adolescents engage in sexual activities before the age of 14 years.^3^ Adolescents are more likely to participate in high-risk sexual behaviours than adults, such as having multiple partners or engaging in sex without using a condom.^4^ Thus, it is unsurprising for the incidence of HIV among adolescents aged 13-19 years in Malaysia to be steadily increase.^3^ According to the 2022 Global AIDS Monitoring Report in Malaysia, there was a rise in sexually transmitted infections (STIs), particularly among adolescents aged 15-24 years.^5^ Overall, adolescents’ sexual behaviours are becoming more prevalent, riskier and associated with numerous negative health consequences, including premarital sex, STIs, teenage pregnancy and unsafe abortions. ^6^

In response to the above mentioned issues, the Ministry of Education (MOE) in Malaysia introduced sexual education in high schools in 1989 and expanded it to elementary schools in 1994.^7^ This curriculum, known as the Reproductive Health and Social Education programme,^8^ integrates sexual and reproductive health (SRH) topics into subjects such as science, biology, religious and moral education and physical education.^7^ However, sexual education remains a taboo topic in Malaysia and difficult to be institutionalised due to public pressure. Despite strong evidence that school-based sexual education helps young people resist pressure to engage in sexual activity, the topic continues to be debated by many parents, teachers, students, the public and the government, with no clear solutions being put forward. Many fear that providing information about sexual matters will spark curiosity and lead to experimentation.

This reflects a societal stigma that needs to be addressed. Comprehensive sex education is not about teaching sexual behaviours, as negatively perceived.

A new approach tailored to the local context should be explored to break the taboo surrounding SRH education. In Malaysia, the typical methods for educating adolescents on SRH include health talks, group discussions, counselling and distribution of pamphlets. These approaches often require trained medical staff and can be time-consuming. Recently, game-based learning has gained popularity and is expanding beyond the classroom setting. It can be delivered online through social media platforms or offline in training sessions. Various technologies have been developed to support the design of gamified courses.^9^ In general, the game-based learning approach shows great potential for SRH education.

Despite its growing popularity in health awareness and promotion programmes, the use of gamified SRH education remains limited in Malaysia. In contrast, countries with similar settings, such as Indonesia, have implemented gamified SRH education more frequently, yielding better learning outcomes.^10^ This approach fosters creative expression and makes learning more enjoyable and satisfying. Game-based approach can be highly effective in increasing adolescent engagement, and most importantly, improving their understanding.^11^ Studies have shown that these games tend to be more popular with adolescent boys, who are often motivated by exploration and gameplay mechanics.^12^ Additionally, SRH topics are generally more comfortable for adolescents when playing games with peers of the same gender, helping them to avoid discomfort.^13^ Therefore, this study aimed to develop and validate a newly gamified SRH module for adolescents. This aligns with the Malaysian government’s endeavour to incorporate comprehensive sexual education into educational institutions with less taboo and rejection.

## Methods

**The development process based on the analysis, design, development, implementation and evaluation (ADDIE) model**

The SRH module for boys *(ReReki)* was developed using the ADDIE model^14^ and concepts from the theory of planned behaviour.^15^ It was designed to provide adolescent boys aged 13-17 years with foundational knowledge in SRH education. This module is particularly important for this age group, as the average age for boys’ sexual debut worldwide ranges from 15 to 17 years, with some boys in Malaysia starting as early as 14 years.^3^ Therefore, this module focused on adolescent boys aged 13-17 years, which is considered as age-appropriate for the Malaysian context.

### Analysis stage

The ADDIE model began with the analysis stage, including assessing needs, clarifying problems and establishing goals. During this stage, the researchers conducted a systematic reviews and meta-synthesis. The keywords used for the search were ‘sexual and reproductive health and adolescent boys’, ‘HIV/AIDS and adolescent boys’, and ‘STD/STI and adolescent boys’. Additionally, local leaflets on gender-related issues, STDs/STIs, and HIV/AIDS were collected. Based on an analysis of the relevant materials, suitable topics were selected to be included in the module.

The content of the *ReReki* module was developed using the threat-coping appraisal framework and reviewed by six panel experts, including public health physicians, family medicine specialists and health educationists. They evaluated five key topics with 29 subtopics. The experts were chosen in regards to their expertise and experience in adolescents’ SRH issues. This stage also involved gathering data through discussions and brainstorming ideas to be included in the module, focusing on what is needed for educators to effectively teach in schools. Subsequently, 20 adolescent boys were invited to test the suitability of six SRH games designed to enhance knowledge, social norms, self-efficacy and permissive attitudes, aligned with the constructs of the theory of planned behaviour.

### Design stage

In the design stage, the learning objectives for the *ReReki* module were outlined; the content was defined; and specific game activities were identified for each module. The evaluation methods for validation were designed. The learning objectives of the module were to (i) increase adolescent boys’ understanding of SRH, including the causes of STIs and STDs, and (ii) enhance their ability to reduce factors that influence their sexual intentions, such as self-efficacy in engaging in sexual activity, social norms surrounding premarital sex and permissive attitudes towards it.

The *ReReki* module was structured into five major topics, presented through six game-based approach activities. The topics were as follows: (i) sexuality in teens, which provided an overview of sexuality; (ii) gender and sexuality, which emphasised understanding the reproductive system and puberty; (iii) sexual health, which covered gender roles, risky behaviours and sexual diseases involving men; (iv) play safe, which promoted abstinence and positive identity development; and (v) law and sexual offenses against children, which addressed legal enforcement on sexual acts. The content of each module was developed based on the learning objectives and the constructs of the theory of planned behaviour as shown in Table 1.

**Table 1:** Outline of the content of the *ReReki* module

Topic	Theory of Planned Behaviours model construct	Objective	Content outline	Game activity
1. Sexuality in teens	Perceived attitude	To educate participants on sexuality.	Explanation of the stage of hormonal changes associated with sexuality, the significance of emotional changes and the role of social psychology in developing a commendable personality.	Let’s Xplore
2. Gender and sexuality	Perceived attitude	To educate participants on SRH life skills and values.	Explanation of human physiology and the evolution of the puberty phase in adolescents.	The RED Zone Box & Portal
3. Sexual health	Perceived behavioural control	To educate participants on the complications of risky sexual behaviours.	Information about adolescent boys’ risky behaviours as well as sexually transmitted diseases.	Message Card 4 U
4. Play Safe	Perceived social norms	To educate participants on the abstinence measures.	Information regarding preventive measures against risky behaviours.	This & That
5. Law and sex offences against children	Perceived behavioural control	To educate participants on the laws that protect adolescents from sexual misconduct.	Explanation regarding laws related to adolescents and sexual offences.	*MONOReReki*

### Development stage

In the development stage, instructions for playing the six games - Let’s X-plore, The Red Zone, Message Card 4 U, Box & Portals, This & That, and *MONOReReki* - were created to achieve the learning objectives. These game elements and instructions were piloted with 20 adolescent boys to ensure that the games were enjoyable and aligned with the objectives, and that the instructions were clear and easy to follow. Their reactions were assessed using a survey form with four questions: (i) How fun was the game?; (ii) Were the messages clear?; (iii) Were the games suitable?; and (iv) What are your suggestions to improve the games?. A 4-point Likert scale was used for the first three questions, while the final question was open-ended. The 20 adolescent boys were further asked about the module contents, with most finding the contents appropriate, motivating and agreeing that the module was relevant as a guide for SRH topics.

### Implementation stage

To ensure that the content of the *ReReki* module was acceptable and provided trustworthy information, the researchers consulted six panel experts to evaluate and determine whether the domains included in the module were suitable for adolescent boys. Expert groups refers to individuals with credibility, experience and expertise. Thus, content experts were selected according to two criteria; (i) they must be a current health/medical practitioners and experts in the field of family medicine, public health (HIV/AIDS), children welfare and sexual behavioural health, and (ii) they must be diverse and well-recognised in their field. The inclusion of six experts for the *ReReki* module as a study sample was in line with the recommendation of having six to nine experts to participate in the evaluation process.

### Evaluation stage

Six panel experts participated in the evaluation process by reviewing, evaluating and providing suggestions for improving the content of the *ReReki* module. The panel rated each of the 29 subtopics on a scale of 1 to 4, with 1 being ‘very irrelevant’ and 4 being ‘very relevant’. The content validity level was calculated using a specific formula,^16^ and a score above 70% was considered indicative of good content validity.^17^ All experts involved in the implementation stage also provided feedback on the modules, leading to further revisions being done based on their comments.


$$\frac{\text{Total expert score}}{\text{Maximum score}}\times 100\%=\text{Content validity achievement}$$

In addition to the survey method, the content validity was assessed using Russel’s model.^17^ In this model, the expert panel evaluated the content validity based on five items; (i) This module’s material is in keeping with the knowledge, abilities, and experience of the intended group; (ii) This module’s content is pertinent and engaging; (iii) This module’s material can help people develop good values; (iv) This module’s content is entirely implementable; and (v) This module’s content can alter behaviours for the better.^17^ The experts rated each item on a scale of 1 to 10, with 1 ‘very irrelevant’ and 10 being ‘very relevant’. According to Russel’s model, the content validity score must exceed 75% to be considered acceptable.


$$\frac{\text{Score obtained}}{\text{The total overall score}}\times 100\%=\text{Content validity achievement}$$

**Figure 1:**
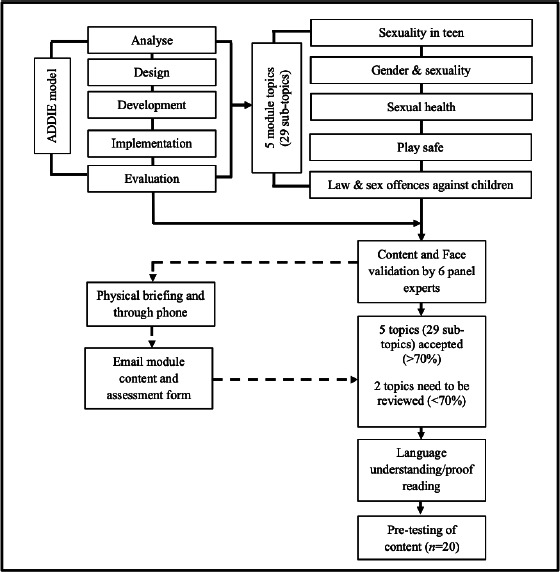
Validation process

Overall, by using the ADDIE model, the researchers fulfilled the following five criteria for a module’s content validity: (i) meeting the population target; (ii) appropriate module implementation method; (iii) adequate time allocated to run the module; (iv) successfully increase in participants’ achievement in the targeted constructs/areas; and (v) positive changes in participants’ attitude. Figure 1 shows the validation process.

## Results

Five main topics and 29 subtopics were developed, aligning well with the theory of planned behaviour. Six panel experts accepted all five topics and 29 subtopics, with an overall content validity score of 81.8% for the survey method and 79.3% for Russell’s model.

The content validity score of 81.8% indicated a strong level of acceptance for the topics and subtopics, ranging from 70% to 90%. This showed that the expert panel approved the list of the SRH topics and subtopics for adolescent boys. However, two subtopics including the sexual response cycle (score=58.3%) and the ID, ego, and superego psychoanalytic theory (score=62.5%) received ratings below 70%. Despite this, the experts agreed to retain these subtopics with the condition that they be refined and enhanced. While the overall content was highly regarded, certain subtopics required refinement in order to ensure that the content aligned with the learning objectives and remained appropriate for the intended audience. The survey method’s content validity score is displayed in Table 2.

**Table 2:** Content validity score based on the survey method

Module’s topic	Sub-topics		Percentage	Expert opinion
Sexuality in teens	1.1	Who are teenagers?	79.2	Accepted
	1.2	What happens during puberty?	87.5	Accepted
	1.3	Emotional, psychological and social	79.2	Accepted
	1.4	Male reproductive system	87.5	Accepted
	1.5	What is sexuality?	83.3	Accepted
	1.6	Why sexuality?	88.3	Accepted
	1.7	What defines sex?	75.0	Accepted
	1.8	Sexual response cycle	58.3	Revised and recommended the introduction of the concept of ‘PPRCS’ (Touch/*Pegang*, Hug/Peluk, Touch/*Ramas*, Kissing/*Cium* and Sex/Seks)
	1.9	Touch	95.8	Accepted
	1.10	Sex arousal	75.0	Accepted
	1.11	Love and sex	75.0	Accepted
Gender and sexuality	2.1	Development of gender	79.2	Accepted
	2.2	Gender Identity	75.0	Accepted
	2.3	Role of gender	83.3	Accepted
	2.4	Gender role and sexual behaviour	75.0	Accepted
	2.5	Sex and gender differences	79.2	Accepted
Sexual health	3.1	Risky sexual behaviour	91.7	Accepted
	3.2	Sexually transmitted diseases	95.8	Accepted
	3.3	Reproductive and sexual health for adolescent boys.	91.7	Accepted
	3.4	What is sex education?	91.7	Accepted
Play safe	4.1	Abstinence	91.7	Accepted
	4.2	Develop a positive self-image	75.0	Accepted
	4.3	Build your confidence	87.5	Accepted
	4.4	Id, ego, super ego	62.5	Revised
	4.5	Religion	95.8	Accepted
	4.6	Condom	87.5	Accepted
Law and sex offences against children	5.1	Penal court	75.0	Accepted
	5.2	Children Act 2011 and Children Act Amended (2016)	79.2	Accepted
	5.3	Sexual Offences Against Children Act 2017	79.2	Accepted
**Overall**			**81.8**	**Accepted**

In Russel’s model, the expert panels rated the content validity of the module as ranging from 75% to 86.7% based on the five key items. Since the minimum content validity rating was beyond 75%, the module’s content could be considered fully implementable. The statement in the module that best aligned with the target group’s knowledge, skills and experience received the highest score of 86.7%. Therefore, the materials could be deemed to have good validity based on the content’s validity having scores above 70%. This indicated that the objectives mentioned earlier were effectively applied when developing the *ReReki* module. Table 3 shows the content validity score.

**Table 3:** Content validity score based on Russell’s model

No.	Item	Percentage	Expert opinion
1.	This module’s material is in keeping with the knowledge, abilities and experience of the intended group.	86.7	Accepted
2.	This module’s content is pertinent and engaging.	80.0	Accepted
3.	This module’s material can help people develop good values.	75.0	Accepted
4.	This module’s content is entirely implementable.	78.3	Accepted
5.	This module’s content can alter behaviours for the better.	76.7	Accepted
**Overall**		**79.3**	

The approved module content was then proofread by two linguistic experts in Malay and English. Phrases and words were refined to match the understanding level of adolescents. For example, ‘anal sex’ was translated as *‘seks dubur* and ‘oral sex’ as ‘*hisap*’ (sucking), terms that adolescents found easier to understand. Finally, the panel experts also provided some suggestions for further improvement, as shown in Table 4.

**Table 4:** Panel experts’ comments for module improvement.

Panel expert	Comment for improvements
Expert 1	Equip with knowledge and ability to make a smart decision for themselves, on anything related to their sexual health. Must be clear what is the goal for boys: i.e., at the end of the day, we don’t want any boys to impregnate any girls because it can lead to other serious/damaging consequences, not to pick up any STIs, never ever become sexual abuser, etc. This topic is relevant, but not too detailed. It can mislead to educating sex instead. Describe each stage. Redundant with puberty topic. I think what you want to highlight here is, as part of puberty, acknowledge that teens do have feelings and it is normal, not something to be ashamed of. Most of them attracted to the opposite sex, but some are attracted to the same sex. How to handle this. The attraction and sexual feeling/desire. Unless your target group is ALL Muslim, then is justified to only mention Islam.
Expert 2	Suggest to explain the term ‘masturbation’. Suggest labelling the findings in the pictures attached, as they might not be able to identify the body parts and what the picture is trying to show. Suggest to remove the statement of ‘living like a monk’ concept, as some religions required their religions to be single. Suggest mentioning all religions against pre-marital sex and advocate sex after marriage.
Expert 3	Suggest to rephrase to ‘Under the Malaysian Law, sex with a girl below 16 years of age is statutory rape regardless of whether consent was given’. Suggest educating teenagers more on self-resilient.
Expert 4	Try to highlight which part the adolescent must know. Try to target this domain. Focus and teach them what domain they must know. Why highlight Western pictures? Why not Malaysian to get some connection between the issues and the adolescent?
Expert 5	Add diversity term and in religion subtopic, make is general and not specific on Islam as a whole. Look at your target audience. Add libido (pheromone).
Expert 6	This statement only refers teen and women/female. Why role of men/male is not included because this module is referring to men/male role?

## Discussion

The *ReReki* SRH module for adolescent boys in Malaysia was developed using the ADDIE instructional design model, resulting in a more comprehensive, authentic and high-quality module tailored to the target audience. The content validity showed good acceptance of the five topics and 29 subtopics, indicating suitability for Malaysian boys aged 13-17 years. The ADDIE model has been used in previous studies to improve the teaching and learning processes.^18^ For instance, it was used to develop reproductive health topics on puberty for deaf students using digital pocketbooks,^18^ a curriculum on LGBT reproductive health employing active learning to address disparities^19^, and the App Law and Reproductive Health initiative aimed at promoting and preventing reproductive health issues.^20^ The SRH *ReReki* module focuses on the SRH of adolescent boys.

Apart from its use in SRH modules, the ADDIE model is widely applied in various learning contexts, including kindergartens, chest radiography, multimedia education, moral education and online language learning. The SRH module demonstrated high validity in practice, meeting the goals of developing students’ critical thinking and moral reasoning skills. Teachers can systematically design their SRH lessons using this module as a guideline, helping educators achieve their teaching objectives and create highly effective courseware. ^21^

The SRH *ReReki* module covers five key topics: (i) sexuality in teens, (ii) gender and sexuality, (iii) sexual health, (iv) play safe, and (v) laws and sexual offences against children, along with 29 subtopics. These contents were based on the theory of planned behaviour, which emphasises perceived attitudes, social norms, behavioural control and sexual intentions.^15^ The gamified module offers several benefits, including the following: (i) increased knowledge of STDs/STIs and HIV/AIDS, (ii) negative attitude toward premarital sex, (iii) perceived social norms of what is being practised and (iv) perceived self-efficacy to control sexual activities. The module is designed for boys aged 13-17 years, aligning with their abilities, experiences and interests, and can be delivered over 5 hours.

To effectively teach adolescent boys about SRH, educators can apply the gamified SRH *ReReki* module as a teaching tool or aid. In the Malaysian context, this module can also serve as supplementary SRH teaching material in secondary schools. Additionally, it can be used by other agencies to enhance their existing sexual health education programmes. Previous research on the development of guidance and counselling modules for preventing risky sexual behaviours in young people has suggested that a module’s validity can be established if it fulfils one of the self-sufficiency criteria. Therefore, guidance on reproductive health for high school students provided by counsellors, is intended to help them maintain their reproductive health and avoid related issues.^22^ Furthermore, the *ReReki* module teaches students how to seek help and support. It provides these boys with factual and useful information, particularly concerning sexual abuse.

The gamified SRH education module covers topics such as the importance of covering one’s genitalia, understanding who is allowed to hug or kiss a child on the cheek, recognising the signs of abuse, identifying situations where abuse might occur, and learning how to refuse and report it.^21^ In addition, the gamified SRH *ReReki* module introduces stages of sexual activity in the form of ‘Hug, Cuddle, Grope, Kiss, and Sex’ or *‘PPRCS - Pegang, Peluk, Raba, Cium* and *Seks*. This content differs from the typical SRH content by offering a clearer breakdown of the sexual process.

Another compelling subtopic of the play safe topic is Freud’s psychoanalytic theory of the id, ego, and superego.^23^ In this topic, adolescent boys are introduced to (i) the ‘id’, representing the instinctual and primitive part of the mind that harbours hidden memories of aggression and sexual desire, (ii) the ‘ego’, which mediates between the desires of the id and superego, and (iii) the ‘superego’, which acts as the moral conscience or compass.^24^ An additional topic included in the module focuses on sex offences involving children, emphasising what adolescents should do if they experience sexual abuse or harassment from inappropriate individuals.

Since SRH is the central focus of this study, the module was validated by subject-matter experts with experience working with adolescents. In this study, the researcher collaborated with social workers specialising in sex offences, health behaviour experts and specialists in family medicine and adolescent health. This approach ensured a broader perspective going beyond sex, disease, and physiology.

## Limitations

While this study successfully addressed its research objectives, it is important to acknowledge certain limitations and challenges. Ethical considerations were particularly significant, as research on SRH education can be perceived as highly sensitive and potentially harmful to adolescents. The contents provided had to be carefully evaluated to ensure that they were safe and did not negatively influence the sexual behaviours of teenagers. This topic in general certainly requires thoughtful discussion, especially when addressing sensitive subjects such as pregnancy prevention, which is especially relevant to adolescent boys seeking preventive measures.

## Conclusion

This study establishes the gamified SRH *ReReki* module for adolescent boys, developed and evaluated empirically using the ADDIE model. The content validity of the ReReki module is confirmed, proving its high quality and reliability. This gamified SRH module can serve as an alternative educational material for SRH education, suitable for use in educational institutions in Malaysia. Its purpose is to promote accurate information and early exposure to safe and beneficial SRH practices, particularly at a time when teenagers are increasingly exposed to misinformation and misconceptions about SRH issues.

## Data sharing statement

The datasets used and/or analysed during the current study and data supporting the findings are available from the corresponding author (Nazrie Saini; nazriesaini@gmail.com) on reasonable request. Raw data were generated at Universiti Malaysia Sarawak.

**How does this paper make a difference in general practice?**

- The findings of this study can guide facilitate sexual healthcare professionals in conducting needs assessment and/or in creating, implementing, and assessing programmes aiming to promote sexual health, particularly in developing countries such as Malaysia. The findings can also assist health authorities in developing a sexual module compatible with the Malaysian culture.
- The findings underscore the importance of comprehensive sexuality education within a salutogenic framework, shifting from disease pathogenesis-based interventions to salutogenesis-based health-promoting interventions. This involves integrating gender perspectives and fostering a comprehensive approach to health promotion.
